# The effects of probiotics plus dietary fiber on antipsychotic-induced weight gain: a randomized clinical trial

**DOI:** 10.1038/s41398-022-01958-2

**Published:** 2022-05-04

**Authors:** Jing Huang, Chenchen Liu, Ye Yang, Dongyu Kang, Jingmei Xiao, Yujun Long, Bing Lang, Xingjie Peng, Weiyan Wang, Xiaoyi Wang, Fangkun Liu, Jingping Zhao, Zhe Shi, Ti-Fei Yuan, Renrong Wu

**Affiliations:** 1 Department of Psychiatry, and National Clinical Research Center for Mental Disorders, The Second Xiangya Hospital of Central South University, 410011 Changsha, Hunan China; 2 Department of Neurosurgery, Xiangya Hospital, Central South University, 410008 Changsha, Hunan China; 3 Key Laboratory for Quality Evaluation of Bulk Herbs of Hunan Province, Hunan University of Chinese Medicine, 410208 Changsha, Hunan China; 4 Shanghai Key Laboratory of Psychotic Disorders, Shanghai Mental Health Center, Shanghai Jiao Tong University School of Medicine, Shanghai, China; 5 Co-innovation Center of Neuroregeneration, Nantong University, Nantong, China; 6 Translational Research Institute of Brain and Brain-Like Intelligence, Shanghai Fourth People’s Hospital Affiliated to Tongji University School of Medicine, Shanghai, China

**Keywords:** Psychiatric disorders, Neuroscience

## Abstract

Probiotics plus dietary fiber has demonstrated efficacy in improving metabolic abnormalities. However, the efficacy of probiotics and dietary fiber as well as their association with microbiota in attenuating antipsychotic-induced weight gain and metabolic disturbance remains poorly understood. Here we analyzed results from the double-blind, randomized, placebo-controlled study to compare and evaluate the effects of probiotics, dietary fiber, and their combination for antipsychotic-induced weight gain in patients with a severe mental disorder. We found that probiotics plus dietary fiber was significantly superior to probiotics alone, dietary fiber only, and the placebo for weight, BMI, and total cholesterol reduction; insulin resistance was worse in the placebo group, with significant increases during the 12-week treatment; probiotics plus dietary fiber significantly reduced weight and prevented further deterioration of metabolic disturbances; and probiotics or dietary fiber alone can prevent further weight gain. We further performed 16 S ribosomal RNA sequencing revealed an increased abundance of microbiota after probiotics plus dietary fiber treatment. Moreover, logistic regression analyses revealed that the higher richness of microbiota was associated with favorable weight loss. These findings suggested that probiotics and dietary fiber co-administration were safe and effective interventions to reduce weight gain in patients treated with antipsychotic medications.

## Introduction

Almost all antipsychotic medications induce weight gain, insulin resistance, and metabolic disturbances [1, 2]. The long-term use of antipsychotics, especially olanzapine, confers a higher risk for weight gain and metabolic disturbances compared with other antipsychotics, which contribute to increased risk for cardiovascular morbidity and premature death [3–5]. Previous studies have indicated that antipsychotic-induced weight gain and metabolic dysfunctions were partly attributable to gut microbiome alterations [6, 7], which can be attenuated by antibiotic administration [8, 9]. Olanzapine-treated rats showed altered fecal microbiota profiles, which were associated with olanzapine-induced metabolic changes [10, 11], suggesting the important role of gut microbiota in antipsychotic-induced weight gain.

Probiotics and dietary fiber are often promoted for various health benefits via independently or synergistically gut microbiota modulation [12]. Probiotics are live microorganisms that confer multiple health benefits to the host [13–15]. Dietary fiber is a nondigestible ingredient that can be fermented by gut microbiota to generate short-chain fatty acids [15], which mediated immune regulation and controlled satiety [16]. Butyrate produced by the gut microbiota through bacterial fermentation of dietary fibers was effective in antioxidant responses, anti-inflammation, and alleviating intestinal barrier dysfunction [17]. As the main source of nutrients for microbiota, dietary fiber plays an important role in shaping the microbiota composition [18]. Previous studies demonstrated the effectiveness of probiotics and/or dietary fiber in weight loss and metabolic improvements in obesity or diabetic populations. The addition of dietary fiber reduced weight gain and improved type 2 diabetes [14]. Kassaian et al. [19] demonstrated that the combination of probiotics and dietary fiber controlled glucose better in pre-diabetic individuals. However, few studies have explored the effects of probiotics and/or dietary fiber in antipsychotic-induced weight gain [20], and the current data are insufficient to conclude their efficacy.

To fill these gaps, the present work aimed to test the hypothesis that probiotics and dietary fiber consumption added to stable treatment of antipsychotic medications would reduce weight gain and other metabolic side effects in patients with a severe mental disorder. Here we analyzed the results from our double-blind, placebo-controlled trial comparing probiotics and dietary fiber alone or in combination for weight gain induced by atypical antipsychotics. Additionally, patients were invited for microbiota screening to characterize microbiota changes and their relationships to metabolism in four groups.

## Patients and methods

### Patients and procedure

**Fig. 1:**
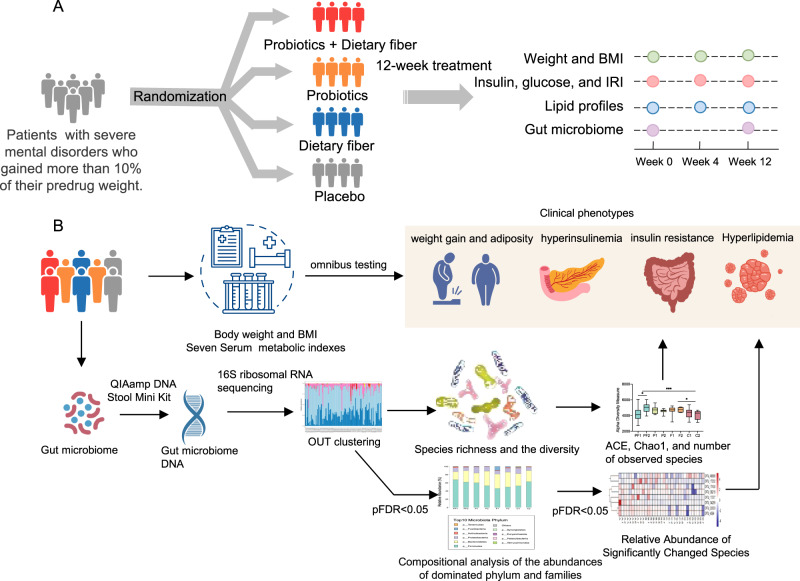
Schematic representation of the main procedure. **A** Patients with a diagnosis of schizophrenia or bipolardisorder were randomized to four treatment groups: probiotics plus dietary fiber, probiotics plus dietary fiberplacebo (probiotics group), dietary fiber plus probiotics placebo (dietary fiber group), or probiotics placebo plusdietary fiber placebo (placebo group). Patients were assessed at screening, baseline, week 4, and week 12 formetabolic-related indexes. **B** Changes in gut microbiota among four groups were examined by 16S ribosomal RNAsequencing. DNA was extracted from stool samples using the QIAampDNA Stool Mini Kit. The hypervariableregions V3-V4 of the 16 ribosomal RNA amplicons were amplified and sequenced by Illumina MiSeq. The speciesrichness and diversity analysis, as well as the compositional analysis were performed among four treatment groups. Logistic regression analyses were conducted to further explore the association of metabolic indexes withthe composition and diversity of gut microbiota.

To assess the effects of probiotics, dietary fiber alone, probiotics plus dietary fiber, and placebo on weight gain and metabolic indexes, we conducted this randomized, placebo-controlled, double-blind clinical trial between August 2019 and June 2021 in the department of psychiatry, the Second Xiangya Hospital. Moreover, we obtained stool samples from participants included and performed 16 S ribosomal RNA sequencing. The study procedure was illustrated in Fig. 1. The trial was approved by the ethics committee of the Second Xiangya hospital, and conducted in accordance with the Declaration of Helsinki of 1975. Written informed consents were obtained from all participants before undergoing examinations. The detailed study protocol has been published previously [21]. Trial registration clinicaltrials.gov Identifier: NCT03379597.

Eligible participants who were receiving atypical antipsychotics with a diagnosis of schizophrenia or bipolar disorder were recruited, based on the criteria of the Diagnostic and Statistical Manual of Mental Disorders Fifth Edition (DSM-5). Inclusion criteria were as follows: (1) aged 18–45; (2) in stable conditions with a total Positive and Negative Syndrome Scale (PANSS) score of ≤60 for patients with schizophrenia or a total Hamilton Depression Scale (HAMD-17) score of ≤7, and a total Young Mania Rating Scale (YMRS) score of <5 for patients with bipolar disorder; (3) with a weight gain of more than 10% after taking atypical antipsychotics; (4) continued to receive their respective atypical antipsychotic medications or antiepileptic drugs throughout the study without change. Exclusive criteria were (1) taking probiotics, fiber supplements, antibiotics, or laxatives in the last 6 weeks; (2) comorbid gastrointestinal diseases, including active peptic ulcer, uncontrolled and recurrent diarrhea, gastrointestinal bleeding, and digestive system neoplastic diseases; (3) unable to complete follow-up.

After the screening period, participants were randomized to four treatment groups in a balanced 2 × 2 factorial design for 12 weeks: probiotics (1680 mg/d) plus dietary fiber (60 g/d), probiotics (1680 mg/d) plus dietary fiber placebo (probiotics group), dietary fiber (60 g/d) plus probiotics placebo (dietary fiber group), or probiotics placebo plus dietary fiber placebo (placebo group). The probiotics formulation used for the study was a live bacteria oral capsule (Bifico, Shanghai Xinyi Pharmaceutical Inc., Shanghai, China). It included a combination of live Lactobacillus, Bifidobacterium, and Enterococcus bacteria with a concentration of 1.7 × 10^9^ CFU/g for Bifidobacterium, 3.8 × 10^8^ CFU/g for Lactobacillus, and 7.8 × 10^8^ CFU/g for Enterococcus. The probiotics placebo was purchased from Shanghai Xinyi Pharmaceutical Inc. as well. The dietary fiber (Perfect Co., Zhongshan, China) included two modified and simplified formulas based on the study from ref. [14], including 10 g HI-FIBER DRINK plus 20 g Extra Herb Powder per packet. Maltodextrin (Qianzhi) was used as the dietary fiber placebo [22]. Participants were instructed to take eight Bifico capsules per day (four capsules after breakfast and four after dinner). The dietary fiber supplement was dissolved in warm water and taken two times per day, one packet per dose together with the probiotics. We declared no conflict of interest with the manufacturers.

Participants were randomized 1:1:1:1 using a computer-generated table in blocks of eight. The randomization and blinding were conducted by the research assistant who was not involved in the study. The randomization letter was sealed and kept separately from the records and data of the study. During the study period, all researchers and participants were blinded to the therapy received.

### Measures

Patients were assessed at screening, baseline, week 4, and week 12 by trained researchers. For the patients with schizophrenia, the overall psychopathology was evaluated by PANSS to evaluate positive and negative symptoms [23]. For patients with bipolar disorder, the symptoms were evaluated by HAMD-17 and YMRS to evaluate depression and mania symptoms, respectively. Treatment Emergent Symptom Scale (TESS) was used for monitoring and evaluating adverse effects. Baseline assessments included demographics, medical history, weight, height, laboratory examinations, PANSS, HAMD-17, YMRS, and TESS. The laboratory examinations included fasting glucose, fasting insulin, lipid profiles, liver and renal function, blood routine test, and electrocardiogram. At each follow-up visit, all baseline evaluations were repeated.

### Gut microbiota analysis

Participants were encouraged to provide stool samples at baseline and week 12, which was not a requirement for completion of the follow-up. Stool samples were collected with a sterile sampling spoon taking out 3–4 g of the middle feces, put in a sterile container, and were then sent for freezing and stored at −80 °C immediately. Changes in gut microbiota were examined by 16 S ribosomal RNA sequencing. DNA was extracted from stool samples using the QIAampDNA Stool Mini Kit (Qiagen). The hypervariable regions V3–V4 of the 16 ribosomal RNA amplicons were amplified and sequenced by Illumina MiSeq. Clean reads from all samples were clustered into OTUs (Operational Taxonomic Units) at 97% similarity and filtered against the Greengenes or Silva reference databases. The sequences were then summarized at the family taxonomic level and analyzed by the ribosomal database project classifier algorithm (http://rdp.cme.msu.edu/).

### Sample size

The sample size was calculated based on the treatment difference in body weight between treatment groups according to Parnell and Reimer’s research [24]. For our study, 136 patients were recruited, including 34 in each group. The completer sample size reflects an effect size of 0.30 to detect the differences between treatment groups, based on 85% power, a two-sided significance level of 5%, and 20% of patients lost to follow-up.

### Statistical analysis

The analyses were performed using the Statistical Package for Social Sciences, version 25.0 (SPSS Inc., Chicago, Illinois). A statistical analysis plan was published before in the protocol [21]. For participants with at least one follow-up visit (118 of 136), the last-observation carried-forward method (intention-to-treat analysis) was employed for efficacy analysis, and all the randomized 136 patients were included for safety analysis. Continuous variables were described using summary statistics (means and standard deviations) and analyzed by ANOVA, categorical variables were described using percentages and analyzed by the χ^2^ test, respectively. The primary and secondary outcomes were compared with ANOVA, with corresponding baseline values as covariates. The omnibus testing was used to test the overall differences among four treatment groups; when the omnibus analysis *p-*value was <0.05, further post-hoc tests were performed to compare the differences between two specific groups. Data normality was evaluated with the Kolmogorov-Smirnov test. All the data were normally distributed. Two-tailed *p*-values ≤0.05 were considered statistically significant.

For Microbiota α-diversity, the richness was evaluated by an abundance-based coverage estimator (ACE) and Chao1. The diversity was evaluated by the observed species, Simpson, and Shannon (multiple comparisons, one-way ANOVA). The rank-based nonparametric Kruskal–Wallis test with FDR correction was used to compare the relative abundances of microbes of four treatment groups; when the overall pFDR was <0.05, further pairwise comparisons by Dunn’s post-hoc tests for multiple comparisons were performed to compare the differences between two specific groups. We performed multivariable logistic regression analysis to investigate associations between gut microbiota changes and metabolic changes including weight, BMI, IRI, insulin, glucose, cholesterol, and HDL-C. Results were presented as odds ratios (ORs) with 95% CIs. Candidate microbiota measures (ACE, Chao1, Observed_species) were classified in quartiles. High species ((above vs below median) presented a high relative abundance of the specific species.

## Results

### Demographics and clinical characteristics

There were 173 patients screened for eligibility, of which 19 patients did not meet the inclusion or met exclusion criteria and 18 patients declined to participate; 136 eligible patients were randomly and equally assigned to four treatment groups. 118 patients had at least one follow-up. Finally, 83.1% of patients (113/136) completed the 12-week treatment. The demographic or clinical characteristics and baseline measurements did not differ significantly among the four groups (Table 1).

**Table 1:** Demographic and clinical characteristics of 118 participants across treatment groups at baseline.

Characteristics	Total (*N* = 118)	Probiotics plus dietary fiber	Probiotics	Dietary fiber	Placebo	Test	*P*
		(*n* = 32)	(*n* = 29)	(*n* = 29)	(*n* = 28)	Statistics^a^	
Age, y	25.55 (24.48–26.63)	24.88 (22.60–27.16)	24.03 (21.98–26.08)	26.76 (24.73–28.80)	26.53 (24.11–28.95)	1.477	0.224
Duration, months	47.05 (37.36–56.74)	47.53 (23.60–71.46)	41.18 (22.17–60.19)	54.88 (38.23–71.53)	44.62 (24.53–64.70)	0.349	0.790
Dose, mg	11.57 (10.60–12.53)	11.41 (9.36–13.45)	12.34 (10.24–14.45)	10.90 (9.14–12.65)	11.64 (9.52–13.77)	0.368	0.776
Weight, kg	70.99 (68.92–73.06)	69.11 (65.52–72.71)	72.44 (67.77–77.12)	70.97 (66.71–75.23)	69.49 (65.71–73.27)	0.439	0.726
BMI, kg/m^2^	27.17 (26.52–27.82)	26.25 (25.02–27.48)	27.41 (26.22–28.60)	27.39 (25.91–28.87)	27.61 (26.18–29.05)	0.882	0.452
Glucose, mmol/L	4.61 (4.48–4.74)	4.80 (4.55–5.05)	4.52 (4.33–4.72)	4.40 (4.19–4.61)	4.67 (4.19–5.15)	1.873	0.137
Insulin, µIU/mL	15.33 (13.94–16.73)	16.75 (13.62–19.87)	16.53 (13.82–19.23)	13.39 (10.94–15.84)	13.35 (10.51–16.19)	1.293	0.280
IRI	3.20 (2.88–3.52)	3.60 (2.89–4.31)	3.32 (2.76–3.87)	2.67 (2.15–3.19)	2.92 (2.13–3.71)	1.493	0.220
Triglyceride, mmol/L	1.71 (1.54–1.88)	1.72 (1.23–1.85)	1.59 (1.22–1.97)	1.86 (1.49–2.22)	1.84 (1.49–2.13)	0.901	0.443
Cholesterol, mmol/L	4.45 (4.33–4.57)	4.43 (4.17–4.70)	4.24 (4.01–4.48)	4.40 (4.18–4.63)	4.66 (4.46–4.86)	2.250	0.086
HDL-C, mmol/L	1.20 (1.15–1.24)	1.25 (1.16–1.35)	1.16 (1.07–1.25)	1.13 (1.03–1.23)	1.24 (1.15–1.34)	1.733	0.163
LDL-C, mmol/L	2.78 (2.68–2.88)	2.70 (2.48–2.92)	2.68 (2.47–2.90)	2.80 (2.64–2.97)	2.93 (2.72–3.13)	1.236	0.299

BMI was calculated as weight in kilograms divided by height in meters squared; IRI was calculated as insulin level (mIU/L) × fasting glucose(mmol/L)/22.5. Data were presented with mean and 95% confidence interval. Dose was calculated as olanzapine equivalent dosage. *P*-values represented the differences in characteristics at baseline across four treatment groups.

*BMI* body mass index, *IRI* insulin resistance index, *HDL-C* high-density lipoprotein cholesterol, *LDL-C* low-density lipoprotein cholesterol.

^a^Test Statistics: analysis of variance for continuous variables.

### Effects of probiotics, dietary fiber, or their combination on weight gain and BMI changes

After 12-week treatment, the weight and BMI decreased significantly in the probiotics plus dietary fiber group and increased remarkably in the placebo group at each follow-up session, but did not change in the probiotics or dietary fiber group (Table 2). Over the 12-week treatment, compared with baseline, weight decreased by 2.36 kg in the probiotics plus dietary fiber group (95% CI, 1.34–3.37 kg), while it increased by 2.63 kg (95% CI, 1.01–4.24 kg) in the placebo group. Similarly, the mean BMI decreased by 0.89 (95% CI, 0.48–1.29) in the probiotics plus dietary fiber group, and increased by 1.03 (95% CI, 0.36–1.70) in the placebo group, but did not change in the probiotics group or the dietary fiber group (Table 3).

**Table 2:** Treatment outcomes of 118 participants across treatment groups at week 4 and week 12.

		Probiotics plus dietary fiber	*P*	Probiotics	*P*	Dietary fiber	*P*	Placebo	*P*
		(*n* = 32)		(*n* = 29)		(*n* = 29)		(*n* = 28)	
Weight, kg									
	Week 4	68.73 (64.95–72.50)	0.325	71.94 (67.34–76.54)	0.102	70.58 (65.89–75.28)	0.891	70.37 (66.59–74.15)	0.013
	Week 12	66.74 (62.77–70.71)	<0.001	72.30 (67.78–76.82)	0.657	70.39 (65.32–75.46)	0.875	71.85 (68.01–75.69)	0.005
BMI, kg/m^2^									
	Week 4	26.12 (24.86–27.38)	0.479	26.99 (25.90–28.08)	0.111	27.47 (25.74–29.20)	0.750	27.41 (26.13–28.69)	0.040
	Week 12	25.37 (24.01–26.73)	<0.001	27.15 (26.02–28.28)	0.761	27.39 (25.50–29.28)	0.793	28.05 (26.72–29.37)	0.007
Insulin, µIU/mL									
	Week 4	14.27 (11.69–16.84)	0.208	16.89 (13.78–20.00)	0.923	15.08 (12.37–17.79)	0.079	15.66 (12.51–18.80)	0.051
	Week 12	14.81 (12.22–17.41)	0.439	17.56 (13.97–21.15)	0.970	14.12 (11.42–16.82)	0.364	17.73 (13.98–21.48)	0.002
IRI									
	Week 4	3.06 (2.46–3.66)	0.224	3.53 (2.83–4.24)	0.992	2.85 (2.33–3.36)	0.230	3.58 (2.78–4.38)	0.023
	Week 12	3.18 (2.56–3.81)	0.462	3.58 (2.83–4.32)	0.961	2.77 (2.24–3.31)	0.478	4.05 (3.13–4.96)	<0.001
Glucose, mmol/L									
	Week 4	4.76 (4.54–4.98)	0.815	4.82 (4.43–5.21)	0.351	4.39 (4.11–4.66)	0.958	5.17 (4.81–5.52)	0.039
	Week 12	4.74 (4.56–4.92)	0.722	4.66 (4.35–4.98)	0.780	4.54 (4.33–4.75)	0.484	5.06 (4.77–5.36)	0.071
Triglyceride, mmol/L									
	Week 4	1.71 (1.26–2.16)	0.561	1.43 (1.16–1.71)	0.284	1.41 (1.12–1.70)	0.734	1.76 (1.40–2.12)	0.875
	Week 12	1.66 (1.41–1.91)	0.635	1.46 (1.23–1.69)	0.280	1.44 (1.19–1.68)	0.779	1.87 (1.54–2.19)	0.338
Cholesterol, mmol/L									
	Week 4	4.21 (3.95–4.47)	0.023	4.35 (3.99–4.71)	0.947	4.53 (4.24–4.81)	0.742	4.78 (4.48–5.07)	0.267
	Week 12	4.16 (3.89–4.42)	0.024	4.42 (4.08–4.76)	0.639	4.51 (4.22–4.79)	0.777	4.76 (4.48–5.05)	0.454
HDL-C, mmol/L									
	Week 4	1.22 (1.13–1.32)	0.573	1.15 (1.05–1.26)	0.948	1.13 (1.04–1.22)	0.833	1.26 (1.14–1.39)	0.566
	Week 12	1.27 (1.17–1.37)	0.810	1.20 (1.10–1.30)	0.238	1.14 (1.07–1.22)	0.994	1.16 (1.05–1.28)	0.011
LDL-C, mmol/L									
	Week 4	2.71 (2.49–2.94)	0.790	2.83 (2.52–3.13)	0.830	2.94 (2.69–3.20)	0.483	2.97 (2.77–3.17)	0.454
	Week 12	2.77 (2.53–3.00)	0.424	2.86 (2.57–3.16)	0.603	2.89 (2.61–3.16)	0.818	3.00 (2.80–3.21)	0.432

BMI was calculated as weight in kilograms divided by height in meters squared; IRI was calculated as insulin level (mIU/L) × fasting glucose(mmol/L)/22.5. Data were presented with mean and 95% confidence interval. *P*-values represented the differences of main outcomes between each time point and baseline within the treatment group.

*BMI* body mass index, *IRI* insulin resistance index, *HDL-C* high-density lipoprotein cholesterol, *LDL-C* low-density lipoprotein cholesterol.

**Table 3:** The difference between baseline and end point of all treatment outcomes.

	Probiotics + dietary fiber	Probiotics	Dietary fiber	Placebo	*P*^*a*^	Probiotics + dietary fiber vs	Probiotics + dietary fiber vs	Probiotics + dietary fiber vs	Dietary fiber vs	Dietary fiber vs	Probiotics vs
	(*n* = 32)	(*n* = 29)	(*n* = 29)	(*n* = 28)		Dietary fiber	Probiotics	Placebo	Probiotics	Placebo	Placebo
	Mean (95% CI)					*P*^*b*^					
Weight, kg	−2.36	−0.56	−0.34	2.63	<0.001	0.026	0.050	<0.001	0.791	0.007	0.003
	(−3.37 to −1.34)	(−2.02 to 0.91)	(−1.95 to 1.27)	(1.01 to 4.24)							
BMI, kg/m^2^	−0.89	−0.16	−0.17	1.03	<0.001	0.044	0.049	<0.001	0.953	0.004	0.003
	(−1.29 to −0.48)	(−0.67 to 0.36)	(−0.78 to 0.44)	(0.36 to 1.70)							
Insulin, µIU/mL	−1.59	0.27	1.53	4.61	0.018	0.226	0.190	0.002	0.937	0.046	0.057
	(−4.51 to 1.32)	(−2.40 to 2.93)	(−0.98 to 4.03)	(2.02 to 7.20)							
IRI	−0.36	0.07	0.25	1.18	0.005	0.460	0.266	<0.001	0.732	0.007	0.017
	(−1.05 to 0.33)	(−0.58 to 0.73)	(−0.24 to 0.75)	(0.58 to 1.78)							
Glucose, mmol/L	−0.08	0.09	0.12	0.40	0.033	0.636	0.820	0.018	0.497	0.006	0.034
	(−0.30 to 0.15)	(−0.23 to 0.42)	(−0.12 to 0.36)	(0.02 to 0.77)							
Triglyceride, mmol/L	0.11	−0.05	0.04	0.18	0.303	0.995	0.223	0.449	0.242	0.465	0.061
	(−0.16 to 0.38)	(−0.21 to 0.11)	(−0.19 to 0.27)	(−0.10 to 0.46)							
Cholesterol, mmol/L	−0.25	0.10	0.06	0.12	0.033	0.041	0.025	0.006	0.837	0.391	0.505
	(−0.44 to −0.06)	(−0.15 to 0.34)	(−0.14 to 0.25)	(−0.11 to 0.36)							
HDL-C, mmol/L	0.02	0.06	0.00	−0.12	0.024	0.230	0.829	0.010	0.167	0.174	0.007
	(−0.05 to 0.08)	(−0.02 to 0.13)	(−0.08 to 0.08)	(−0.21 to −0.04)							
LDL-C, mmol/L	0.10	0.08	0.05	0.13	0.885	0.915	0.907	0.515	0.829	0.460	0.597
	(−0.08 to 0.27)	(−0.11 to 0.28)	(−0.14 to 0.24)	(−0.10 to 0.35)							

BMI was calculated as weight in kilograms divided by height in meters squared; IRI was calculated as insulin level (mIU/L) × fasting glucose(mmol/L)/22.5. Data were presented with mean and 95% confidence interval.

*P*^*a*^: *p*-value for the overall differences among four groups was tested initially by the omnibus analysis and based primarily on ANCOVA with baseline levels of the variables as covariates. Follow-up pairwise comparisons (*P*^*b*^) were performed when the overall omnibus analysis *p*-value was significant.

*BMI* body mass index, *IRI* insulin resistance index, *HDL-C* high-density lipoprotein cholesterol, *LDL-C* low-density lipoprotein cholesterol.

For the mean changes of weight and BMI from baseline to week 12, specific pairwise comparisons of the treatments groups indicated that (1) Probiotics plus dietary fiber was significantly superior to probiotics, dietary fiber, and placebo; (2) Probiotics alone or dietary fiber only was significantly superior to placebo; (3) No significant difference was observed between the probiotics group and dietary fiber group.

### Effects of probiotics, dietary fiber, or their combination on insulin, IRI, glucose, and lipid profiles

The insulin and IRI levels increased significantly at week 12 in the placebo group (*p* = 0.002 and <0.001, respectively), but did not change in the other three treatment groups during the follow-up period. Over the 12-week treatment, patients who received a placebo had a significantly increased insulin level of 4.61 µIU/mL (95% CI, 2.02–7.20 µIU/mL); and a relative increase in IRI of 1.18 (95% CI, 0.58–1.78). Moreover, there was a significant decrease in HDL-C in the placebo group at week 12 (*p* = 0.011). Continuous reduction of cholesterol was observed in the probiotics plus dietary fiber group at week 4 and week 12 (*p* = 0.023 and 0.024, respectively). Indexes related to glucose and other lipid metabolism in the four groups did not change significantly over time (Tables 2 and 3).

For the mean changes of insulin, fasting glucose, and lipid profiles for the four treatment groups from baseline to week 12, the administration of probiotics plus dietary fiber was significantly superior to placebo on insulin, IRI, cholesterol, and HDL-C; was superior to probiotic alone and dietary fiber only on cholesterol. Probiotics alone was superior to placebo on IRI and HDL-C. Dietary fiber only was superior to placebo on insulin and IRI. No significant differences in changes in insulin, IRI, glucose, and lipid profiles were observed between probiotics alone and dietary fiber only.

Excluding constipation, there were no significant differences in the adverse effects and no serious adverse events reported among four treatment groups. Compared with other three groups, the frequency of constipation was higher in the placebo group (*p* = 0.02). The most frequently reported adverse events were somnolence (*n* = 19, 14.0%), constipation (*n* = 18, 13.2%), hypoactivity (*n* = 14, 10.3%), nausea/vomiting (*n* = 5, 3.7%), and abnormal liver function (*n* = 3, 2.2%). Supplemental Table 1 presented adverse events in the entire sample.

### Changes in gut microbiota

**Fig. 2:**
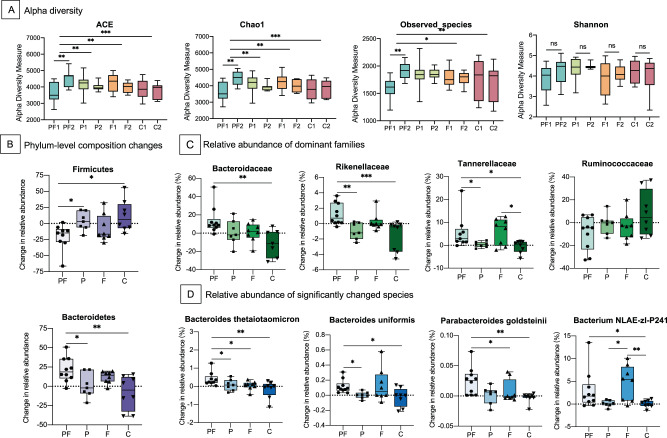
Gut microbiota changes in four treatment groups. **A** Alpha diversity evaluated by richness (abundance-basedcoverage estimator [ACE] and Chao1), Observed species, and diversity (Shannon). Boxes indicate interquartile range, lines indicate medians, and whiskers represent range. **B** Changes in phylum-level composition (Firmicutes and Bacteroidetes) across four treatment groups. **C** Changes in relative abundance of dominant families acrossfour treatment groups. **D** Relative abundance of significantly changed species across four groups. *p < 0.05, **p < 0.01,***p < 0.001.

During the 12-week period, 62 patients provided at least one stool sample, a total of 33 patients who provided stool samples at baseline and at week 12 were included in the 16 S rRNA sequencing. After 12-week treatment, the abundance-based coverage estimator (ACE), Chao1, and the number of observed species increased significantly in the probiotics plus dietary fiber group, while they did not change in the other three groups (Fig. 2A, Supplemental Tables 2–3).

Furthermore, we performed a compositional analysis of the abundances of the top 10 phylum and families. For phylum, a significantly decreased abundance of Firmicutes and increased abundance of Bacteroidetes were observed in the probiotics plus dietary fiber group, which is associated with the improvement of obesity [25, 26] (Fig. 2B). For families, the combination of probiotics and dietary fiber was associated with an increased abundance of Bacteroidaceae, Rikenellaceae, and Tannerellaceae compared with the placebo group (Fig. 1C). Significantly changed species across four treatment groups were summarized in Supplemental Fig. 1. The increased abundances of Bacteroides thetaiotaomicron, Bacteroides uniformis, and Parabacteroides goldsteinii were observed, which have been reported to decrease in obese individuals and its increase alleviated weight gain and adiposity in mice [27, 28] (Fig. 2C and Supplemental Fig. 2, Supplemental Tables 4 and 5).

To further explore the association of metabolic indexes with the composition and diversity of gut microbiota, we performed logistic regression analyses (Supplemental Table 6). The higher richness of microbiota was associated with a decrease in weight (OR for Chao1, 0.37 per quartile increase, 95%CI, 0.15–0.87; OR for ACE, 0.45 per quartile increase, 95%CI, 0.20–0.98); A decreased risk of cholesterol increase was also observed. Higher relative abundance of Parabacteroides goldsteinii was associated with 96% decrease in risk of high IRI (OR, 0.04; 95% CI, 0.002–0.85) and 95% decrease in risk of high cholesterol (OR, 0.05; 95% CI, 0.003–0.74).

## Discussion

The present study demonstrated that probiotics plus dietary fiber was effective in ameliorating antipsychotic-induced weight gain in patients with a severe mental disorder. Patients receiving probiotics plus dietary fiber had a 2.36 kg decrease in weight compared with a 2.63 kg increase in weight in the placebo group. There were also benefits from probiotics plus dietary fiber in the secondary outcomes, including insulin, IRI, fasting glucose, cholesterol, and HDL-C. Although the majority of second outcomes did not change after the 12-week probiotics plus dietary fiber treatment, compared with placebo, probiotics plus dietary fiber could prevent further deterioration of insulin, IRI, and HDL-C induced by antipsychotics. Moreover, the results suggested that probiotics or dietary fiber alone can prevent further weight accretion, IRI, and lipid disturbance deterioration, which continued to deteriorate in the placebo group.

Although there were few findings of probiotics plus dietary fiber on weight gain and metabolic disturbance, much evidence has supported the benefits of probiotics or dietary fiber on obesity and metabolic syndrome. For probiotics, a double-blind RCT that enrolled 57 pregnant women with diet-controlled gestational diabetes mellitus, suggested that probiotics supplements lowered fasting glucose and IRI [29]. In schizophrenia patients, probiotics plus Vitamin D improved clinical symptoms and metabolic dysfunctions [30]. For dietary fiber, in a large study of young adults, Ludwig et al. [31] reported that dietary fiber had protective effects on obesity and cardiovascular diseases by lowering insulin levels. In olanzapine-induced obese rats, Kao et al. [32] found that the inclusion of B-GOS® (kind of dietary fiber) attenuated weight gain, which was in contrast to their research in schizophrenia patients [20].

In the present study, the effects of attenuating weight gain and metabolic disturbances were more evident in the probiotics plus prebiotics group, suggesting that probiotics and dietary fiber may have synergistic effects on the observed improvements. Krumbeck et al. [33] found that probiotics and galactooligosaccharides (kind of dietary fiber) improved colonic permeability but showed no synergism. Emerging evidence has also indicated a synergistic effect of prebiotics and probiotics on host health [34, 35]; A randomized controlled single-blind pilot trial observed the symbiotic effect of probiotic and fructo-oligosaccharide (dietary fiber) on lipid profiles in healthy young volunteers [36]. In our study, the total cholesterol was significantly improved in the combination group, while no significant reduction of LDL-C was observed. Another RCT conducted by Stenman et al. [37] found that the combination of probiotics with fiber reduced waist circumference in overweight and obese adults after 6 months of treatment, while probiotics alone had no effect compared to the placebo.

Previous studies suggested significant shifts towards an “obesogenic” bacterial profile in the human gut microbiota following the use of atypical antipsychotics. A possible modulatory effect of probiotics plus dietary fiber on gut microbiota was observed. In a cohort that assessed the gut microbial composition of 123 non-obese and 169 obese individuals, individuals with a lower bacterial richness correlated with more adiposity, IRI, and dyslipidemia [38]. Patients receiving probiotics plus dietary fiber also displayed favorable changes in the composition of gut microbiota, with a higher Bacteroidetes/ Firmicutes ratio compared with baseline, which was in line with previous studies [25, 39]. The increased abundance of Rikenellaceae in the probiotics plus dietary fiber group was found to be positively associated with leptin levels and participated in weight regulation [40, 41]. Tannerellaceae was thought one of the core rutin-selected bacterial taxa, which exhibited anti-hyperglycemia, dyslipidemia, and cardiovascular disorders [42]. A reduced abundance of Bacteroides thetaiotaomicron was observed in obese individuals, which can be reversed by weight-loss intervention [27]. Our present findings were consistent with this study, probiotics plus dietary fiber supplements induced weight loss and the increased abundance of Bacteroides thetaiotaomicron compared with the placebo.

## Strengths and limitations

To the best of our knowledge, this is the first RCT trial investigating the effect of probiotics and dietary fiber on antipsychotics-induced weight gain. In this study, a balanced 2 × 2 factorial design was used to compare the efficacy of probiotics, dietary fiber, and the combination on weight attenuation and metabolic improvements. Detailed gut microbiota analysis was also included to compare the bacteria differences across groups.

Nevertheless, the study has some limitations. First, we did not evaluate the long-term effects of the treatments after a 12-week treatment period, although most patients visited the out-patients clinic regularly. Second, despite the adjustment of covariates, we could not rule out the influence of dietary structure, exercise habits, or other unmeasured confounders. Third, due to the small sample size of stool samples, the preliminary 16 S ribosomal RNA analysis results need further validation. Fourth, although the results were inconsistent with those observed in other populations, this study was conducted in south-central China. Considering the possible regional variation, further validation in large and more diverse populations is needed.

## Conclusions

The present study found the combination of probiotics and dietary fiber induced significant weight loss, probiotics or dietary fiber alone can prevent further weight gain, while the placebo group had sustained remarkable weight growth. Furthermore, the addition of probiotics and/or dietary fiber helped to remain insulin sensitivity, while the placebo had a significantly worse IRI. The results suggested that probiotics and dietary fiber may be effective and safe interventions to improve weight gain and metabolic disturbance induced by antipsychotics in patients with severe mental disorders.

## Supplementary information

Supplmental file

supplementary figure and table legends.docx

Supplement 1. Trial Protocol

Supplement 2 Consort Checklist

Supplement 3. Data Sharing Statement.
