# Supplementary material for: The effects of probiotics plus dietary fiber on antipsychotic-induced weight gain: a randomized clinical trial
Source: Transl Psychiatry. 2022 May 4;12:185. doi: 10.1038/s41398-022-01958-2 (PMC9068806; doi:10.1038/s41398-022-01958-2)
Supplement: Supplementary file 1 — Supplmental file [file 41398_2022_1958_MOESM1_ESM.docx]

Supplemental Figure 1. Flowchart of Study Participants.

**
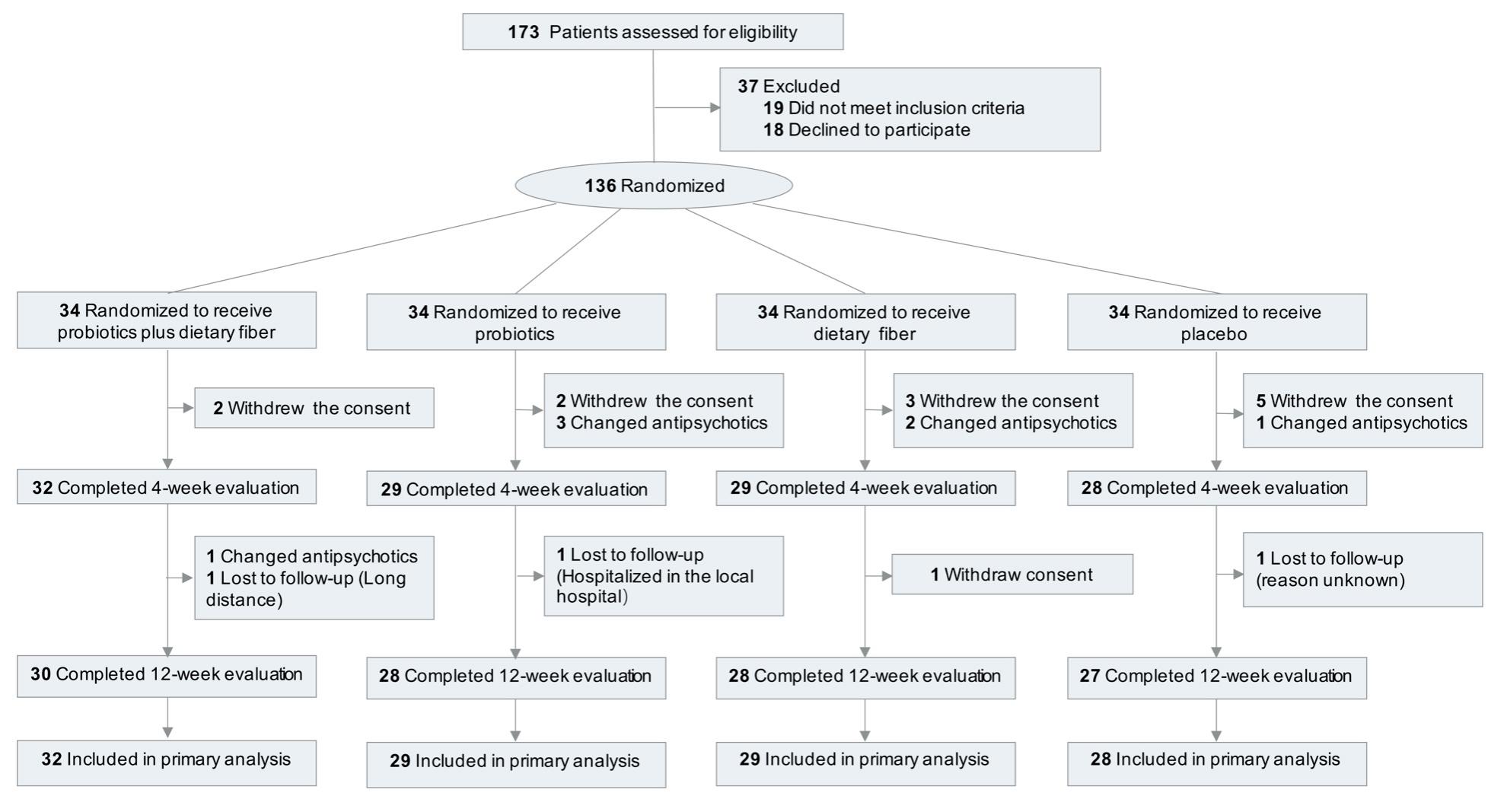
**

Supplemental Figure 2. Relative Abundance of Significantly Changed Species Across Four Treatment Groups.


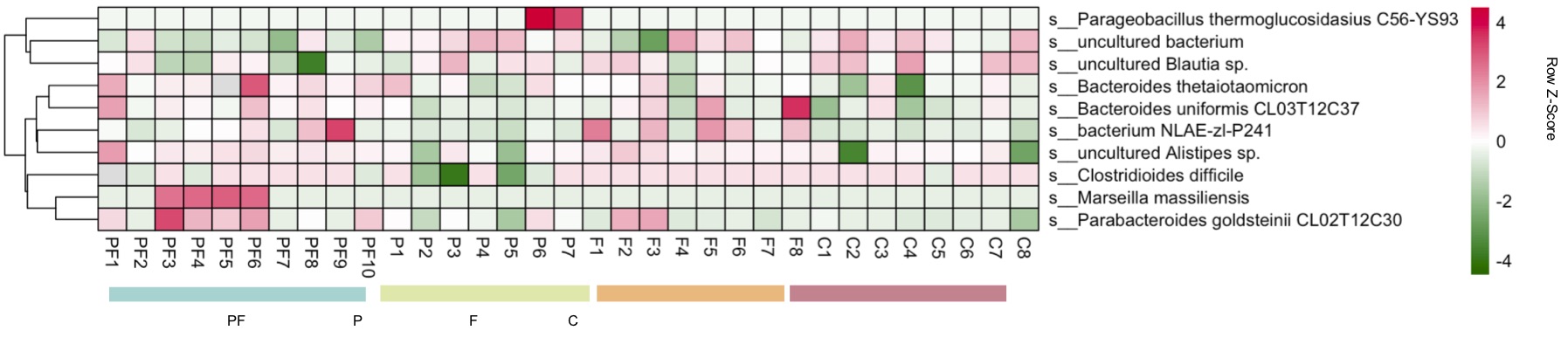


PF represented the probiotics plus dietary fiber group, N=10.

P represented the probiotics group, N=7.

F represented the dietary fiber group, N=8.

C represented the placebo group, N=8.

Supplemental Table 1. Adverse Effects by Treatment Groups.

|  | | No. (%) of Participants | | | |  |  |
| --- | --- | --- | --- | --- | --- | --- | --- |
| Adverse Effect | Probiotics + Dietary fiber (n=34) | | Probiotics  (n=34) | Dietary fiber  (n=34) | Placebo (n=34) | χ^2^ Test^a^ | *P* value |
| Hypoactivity | 4 (11.8) | | 4 (11.8) | 4 (11.8) | 2 (5.9) | 0.96 | 0.81 |
| Somnolence | 3 (8.8) | | 6 (17.6) | 4 (11.8) | 6 (17.6) | 1.24 | 0.74 |
| Abnormal liver function | 1 (2.9) | | 1 (2.9) | 1 (2.9) | 0 (0.0) | 1.02 | 0.8 |
| Constipation | 3 (8.8) | | 2 (5.9) | 3 (8.8) | 10 (29.4) | 10.50 | 0.02 |
| Nausea //Vomiting | 1 (2.9) | | 2 (5.9) | 0 (0.0) | 2 (5.9) | 2.28 | 0.52 |
| Headache | 1 (2.9) | | 0 (0.0) | 0 (0.0) | 0 (0.0) | 3.02 | 0.39 |

^a^χ^2^ Test among four treatment groups.

Supplemental Table 2. Comparison of α-diversity in Four Treatment Groups Between Baseline and Week 12.

|  | Probiotics plus dietary fiber (PF)  N=10 | | | | Probiotics (P)  N=7 | | Dietary fiber (D)  N=8 | | | | Placebo (C)  N=8 | |  |
| --- | --- | --- | --- | --- | --- | --- | --- | --- | --- | --- | --- | --- | --- |
| α-diversity | t *P* | |  | t | | *P* | | t | *P* | t | | *P* | |
| ACE | -3.649 | 0.002 | | 0.590 | | 0.566 | | 1.205 | 0.248 | 0.337 | | 0.741 | |
| Chao 1 | -3.599 | 0.002 | | 0.641 | | 0.534 | | 1.040 | 0.316 | -0.069 | | 0.946 | |
| Observed Species | -3.812 | 0.001 | | -0.131 | | 0.898 | | 0.253 | 0.804 | 0.410 | | 0.688 | |
| Simpson | -0.746 | 0.465 | | -1.024 | | 0.326 | | -1.326 | 0.221 | 0.435 | | 0.670 | |
| Shannon | 0.958 | 0.226 | | -0.627 | | 0.542 | | -0.697 | 0.497 | 0.451 | | 0.659 | |

*P* values represented the differences of gut bacteria between week 12 and baseline within the treatment group.

Supplemental Table 3. Comparison of α-diversity Across Treatment Groups.

| α-diversity  (*P^a^* value) | Probiotics +  dietary fiber  vs  Dietary Fiber | Probiotics +  dietary fiber  vs  Probiotics | Probiotics +  dietary fiber  vs  Placebo | Dietary fiber  vs  Probiotics | Dietary fiber  vs  Placebo | Probiotics  vs  Placebo | *P^b^* |
| --- | --- | --- | --- | --- | --- | --- | --- |
| ACE | 0.002 | <0.001 | <0.001 | 0.804 | 0.586 | 0.438 | <0.001 |
| Chao 1 | 0.004 | <0.001 | <0.001 | 0.957 | 0.78 | 0.824 | 0.003 |
| Observed Species | 0.049 | 0.202 | 0.007 | 0.52 | 0.432 | 0.168 | 0.046 |
| Simpson | 0.696 | 0.552 | 0.552 | 0.666 | 0.366 | 0.178 | 0.565 |
| Shannon | 0.654 | 0.763 | 0.287 | 0.491 | 0.546 | 0.200 | 0.572 |

*P^a^* represents the differences from the baseline to week 12 between groups.

*P^b^* represents the overall differences across four treatment groups.

Supplemental Table 4. Median Relative Abundance of Top 10 Phylum and Families in Fecal Microbiota of Patients in Four Treatment Groups.

| **Phylum** | Probiotics plus dietary fiber (PF)  N=10 | | | | Probiotics (P)  N=7 | | Dietary fiber (D)  N=8 | | | | Placebo (C)  N=8 | |  |
| --- | --- | --- | --- | --- | --- | --- | --- | --- | --- | --- | --- | --- | --- |
| *Family* | Mann-Whitney U *P* | |  | Mann-Whitney U | | *P* | | Mann-Whitney U | *P* | Mann-Whitney U | | *P* | |
| **Firmicutes** | 9 | 0.005 | | 29 | | 0.565 | | 17 | 0.115 | 42 | | 0.294 | |
| *Ruminococcaceae* | 36 | 0.290 | | 24 | | 0.949 | | 26 | 0.529 | 43 | | 0.248 | |
| *Lachnospiraceae* | 36 | 0.290 | | 27 | | 0.749 | | 26 | 0.529 | 34 | | 0.834 | |
| *Veillonellaceae* | 52 | 0.880 | | 23 | | 0.848 | | 33 | 0.916 | 47 | | 0.115 | |
| *Peptostreptococcaceae* | 15 | 0.008 | | 23 | | 0.848 | | 22 | 0.294 | 23 | | 0.345 | |
| **Bacteroidetes** | 92 | 0.001 | | 27 | | 0.749 | | 55 | 0.016 | 25 | | 0.462 | |
| *Bacteroidaceae* | 90 | 0.002 | | 24 | | 0.949 | | 35 | 0.753 | 17 | | 0.338 | |
| *Prevotellaceae* | 62 | 0.364 | | 29 | | 0.565 | | 41 | 0.345 | 28 | | 0.674 | |
| *Tannerellaceae* | 66 | 0.021 | | 29 | | 0.565 | | 57 | 0.009 | 28 | | 0.674 | |
| *Rikenellaceae* | 73 | 0.082 | | 19 | | 0.482 | | 28 | 0.674 | 15 | | 0.074 | |
| **Proteobacteria** | 35 | 0.257 | | 25 | | 0.949 | | 27 | 0.600 | 28 | | 0.674 | |
| *Enterobacteriaceae* | 44 | 0.650 | | 21 | | 0.655 | | 30 | 0.834 | 30 | | 0.834 | |
| **Fusobacteria** | 43 | 0.595 | | 16 | | 0.277 | | 21 | 0.248 | 24 | | 0.401 | |
| *Fusobacteriaceae* | 43 | 0.595 | | 16 | | 0.277 | | 21 | 0.248 | 24 | | 0.401 | |
| **Actinobacteria** | 38 | 0.364 | | 29 | | 0.565 | | 43 | 0.248 | 44 | | 0.208 | |
| **Tenericutes** | 37 | 0.326 | | 31 | | 0.406 | | 28 | 0.610 | 37 | | 0.6 | |
| **Verrucomicrobia** | 51 | 0.940 | | 29 | | 0.503 | | 31 | 0.872 | 30 | | 0.834 | |
| **Patescibacteria** | 39 | 0.402 | | 25 | | 0.949 | | 23 | 0.341 | 47 | | 0.112 | |
| **Synergistetes** | 41 | 0.495 | | 19 | | 0.482 | | 33 | 0.916 | 31 | | 0.916 | |
| **Euryarchaeota** | 45 | 0.67 | | 25 | | 0.917 | | 40 | 0.144 | 34 | | 0.783 | |

Wilcoxon matched-pairs signed rank test

*P* values represented the differences of gut bacteria between week 12 and baseline within the treatment group.

Supplemental Table 5. Comparison of Top 10 Phylum and Families in Fecal Microbiota of Patients across Four Treatment Groups.

| **Phylum**  *Family*  (*Pairwise pFDR*) | Overall  pFDR | Probiotics +  dietary fiber  vs  Dietary fiber | Probiotics +  dietary fiber  vs  Probiotics | Probiotics +  dietary fiber  vs  Placebo | Dietary fiber  vs  Probiotics | Dietary fiber  vs  Placebo | Probiotics  vs  Placebo |
| --- | --- | --- | --- | --- | --- | --- | --- |
| **Firmicutes** | 0.033 | 0.452 | 0.028 | 0.010 | 0.150 | 0.079 | 0.794 |
| *Ruminococcaceae* | 0.390 | - | - | - | - | - | - |
| *Lachnospiraceae* | 0.811 | - | - | - | - | - | - |
| *Veillonellaceae* | 0.220 | - | - | - | - | - | - |
| *Peptostreptococcaceae* | 0.442 | - | - | - | - | - | - |
| **Bacteroidetes** | 0.017 | 0.316 | 0.028 | 0.003 | 0.242 | 0.066 | 0.546 |
| *Bacteroidaceae* | 0.019 | 0.095 | 0.073 | 0.002 | 0.857 | 0.157 | 0.231 |
| *Prevotellaceae* | 0.338 | - | - | - | - | - | - |
| *Tannerellaceae* | 0.029 | 0.901 | 0.037 | 0.020 | 0.055 | 0.033 | 0.886 |
| *Rikenellaceae* | 0.004 | 0.128 | 0.007 | 0.001 | 0.232 | 0.093 | 0.669 |
| **Proteobacteria** | 0.950 | - | - | - | - | - | - |
| *Enterobacteriaceae* | 0.983 | - | - | - | - | - | - |
| **Fusobacteria** | 0.147 | - | - | - | - | - | - |
| *Fusobacteriaceae* | 0.147 | - | - | - | - | - | - |
| **Actinobacteria** | 0.118 | - | - | - | - | - | - |
| **Tenericutes** | 0.112 | - | - | - | - | - | - |
| **Verrucomicrobia** | 0.925 | - | - | - | - | - | - |
| **Patescibacteria** | 0.258 | - | - | - | - | - | - |
| **Synergistetes** | 0.537 | - | - | - | - | - | - |
| **Euryarchaeota** | 0.662 | - | - | - | - | - | - |

The rank-based nonparametric Kruskal–Wallis test with FDR correction was used for the overall comparisons of four treatment groups; when the overall *p*FDR was <0.05, further pairwise comparisons by Dunn’s post-hoc tests for multiple comparisons were performed to compare the differences between two specific groups.

Supplemental Table 6. Association of Metabolic Indexes and Key Microbiota Measures

|  | Weight | BMI | IRI | Insulin | Glucose | Cholesterol | HDL-C |
| --- | --- | --- | --- | --- | --- | --- | --- |
| Microbiota Measures | OR (95%CI) | OR (95%CI) | OR (95%CI) | OR (95%CI) | OR (95%CI) | OR (95%CI) | OR (95%CI) |
| Chao1  (per quartile increase) | 0.37 (0.15-0.87) | 0.37 (0.15-0.87) | 0.47 (0.21-1.06) | 0.78 (0.34-1.78) | 0.72 (0.33-1.56) | 0.25 (0.09-0.75) | 1.37 (0.71-2.68) |
|  |  |  |  |  |  |  |  |
| ACE  (per quartile increase) | 0.45 (0.20-0.98) | 0.45 (0.20-0.98) | 0.60 (0.27-1.29) | 1.06 (0.48-2.34) | 0.71 (0.33-1.55) | 0.30 (0.11-0.82) | 1.11 (0.58-2.14) |
|  |  |  |  |  |  |  |  |
| Observed_species  (per quartile increase) | 0.61 (0.30-1.26) | 0.61 (0.30-1.26) | 0.55 (0.25-1.21) | 0.65 (0.28-1.54) | 0.87 (0.40-1.88) | 0.50 (0.22-1.12) | 1.57 (0.79-3.13) |
|  |  |  |  |  |  |  |  |
| dFirmicutes | 0.99 (0.21-4.55) | 0.99 (0.21-4.55) | 4.91 (0.87-27.52) | 4.22 (0.58-30.66) | 10.95 (1.26-94.95) | 1.62 (0.34-7.86) | 3.62 (0.77-17.02) |
| dBacteroidetes | 0.39 (0.08-1.91) | 0.39 (0.08-1.91) | 0.25 (0.04-1.41) | 0.14 (0.02-1.18) | 0.30 (0.05-1.92) | 0.46 (0.09-2.31) | 1.16 (0.27-5.01) |
|  |  |  |  |  |  |  |  |
| Parabacteroides goldsteinii  (above median) | 0.20 (0.03-1.34) | 0.20 (0.03-1.34) | 0.04 (0.002-0.85) | 0.06 (0.004-1.15) | 0.12 (0.01-1.14) | 0.05 (0.003-0.74) | 1.47 (0.30-7.23) |
|  |  |  |  |  |  |  |  |
| Bacteroides uniformis  (above median) | 0.72 (0.15-3.49) | 0.72 (0.15-3.49) | 0.59 (0.11-3.27) | 0.75 (0.12-4.84) | 0.09 (0.007-1.16) | 0.53 (0.10-2.99) | 1.26 (0.28-5.73) |
|  |  |  |  |  |  |  |  |
| Bacteroides thetaiotaomicron  (above median) | 0.33 (0.07-1.63) | 0.33 (0.07-1.63) | 0.15 (0.02-1.09) | 0.14 (0.02-1.24) | 0.27 (0.04-1.93) | 0.31 (0.05-1.84) | 1.54 (0.35-6.90) |

Abbreviations:OR, odds ratio.

Candidate microbiota measures (ACE, Chao1, Observed species) were classified in quartiles.

High species ((above vs below median) presented high relative abundance of the specific species.
