# Supplementary material for: The effects of probiotics plus dietary fiber on antipsychotic-induced weight gain: a randomized clinical trial
Source: Transl Psychiatry. 2022 May 4;12:185. doi: 10.1038/s41398-022-01958-2 (PMC9068806; doi:10.1038/s41398-022-01958-2)
Supplement: Supplementary file 2 — supplementary figure and table legends.docx [file 41398_2022_1958_MOESM2_ESM.docx]

**supplementary figure and table legends**

Supplemental Figure 1. Flowchart of study participants.

Supplemental Figure 2. Relative abundance of significantly changed species across four treatment groups.

Supplemental Table 1. Adverse effects by treatment groups.

Supplemental Table 2. Comparison of α-diversity in four treatment groups between baseline and week 12.

Supplemental Table 3. Comparison of α-diversity across treatment groups.

Supplemental Table 4. Median relative abundance of top 10 Phylum and Families in fecal microbiota of patients in four treatment groups.

Supplemental Table 5. Comparison of top 10 Phylum and Families in fecal microbiota of patients across four treatment groups.

Supplemental Table 6. Association of metabolic indexes and key microbiota measures.
