# Supplementary material for: The effects of probiotics plus dietary fiber on antipsychotic-induced weight gain: a randomized clinical trial
Source: Transl Psychiatry. 2022 May 4;12:185. doi: 10.1038/s41398-022-01958-2 (PMC9068806; doi:10.1038/s41398-022-01958-2)
Supplement: Supplementary file 5 — Supplement 3. Data Sharing Statement. [file 41398_2022_1958_MOESM5_ESM.docx]

**Data Sharing Statement**

**Data**

**Data available:** Yes

**Data types:** Deidentified participant data

**How to access data:** We do not have a repository for the data online at present. Anonymized data is available upon reasonable request including a short study protocol. The National Clinical Research Center for Mental Disorders will evaluate all requests. My email address is wurenrong@csu.edu.cn

**When available:** With publication

**Supporting Documents**

**Document types:** Statistical/analytic code, Informed consent form

**How to access documents:** wurenrong@csu.edu.cn

**When available:** With publication

**Additional Information**

**Who can access the data:** Anonymized data is available upon reasonable request including a short study protocol. The National Clinical Research Center for Mental Disorders will evaluate all requests.

**Types of analyses:** Anonymized data

**Mechanisms of data availability:** Anonymized data is available upon reasonable request including a short study protocol. The National Clinical Research Center for Mental Disorders will evaluate all requests.
